# Prevalence, Awareness, and Treatment of Hypertension in Patients with Type 1 Diabetes: A Nationwide Multicenter Study in Brazil

**DOI:** 10.1155/2013/565263

**Published:** 2013-03-04

**Authors:** Marilia B. Gomes, Lucianne Righeti Monteiro Tannus, Alessandra Saldanha de Mattos Matheus, Roberta Arnoldi Cobas, Catia C. Sousa Palma, Aline Tiemi Kano Silva, Carlos Antonio Negrato, Sergio Atala Dib, Melanie Rodacki, João Soares Felício, Luis Henrique Canani

**Affiliations:** ^1^Department of Internal Medicine, Diabetes Unit, State University of Rio de Janeiro, 20551-030 Rio de Janeiro, RJ, Brazil; ^2^Bauru's Diabetics Association, 17010-130 Bauru, SP, Brazil; ^3^Diabetes Unit, Federal University of São Paulo State, 04021-001 São Paulo, SP, Brazil; ^4^Federal University of Rio de Janeiro, 21941-901 Rio de Janeiro, RJ, Brazil; ^5^Federal University Hospital João Barreto, 66073-000 Belém, PA, Brazil; ^6^Federal University Hospital of Porto Alegre, 90035-903 Porto Alegre, RS, Brazil

## Abstract

*Objective*. This study evaluated the prevalence, awareness, and type of treatment for hypertension in Brazil in patients with type 1 diabetes (T1D). *Methods*. This was a cross-sectional, multicenter study that was conducted from December 2008 to December 2010 in 28 public clinics located in 20 Brazilian cities. *Results*. A total of 3,591 patients were studied, 56% female, average age 21.2 ± 11.7 years, with a median duration of diabetes 9.6 ± 8.1 years. Blood pressure levels were available for a total of 3,323 patients and 689 (19.2%) patients were hypertensive. Hypertensive patients were older, exhibited longer duration of diabetes, and had higher body mass index (BMI), total cholesterol, triglycerides, and LDL-C values (*P* < 0.001, for all comparisons), but only 370 (53.7%) received treatment. Patient awareness of hypertension was documented in 453 (65.5%) patients. However, only 76 (22.9%) of the treated patients attained the target systolic (sBP) and diastolic blood pressures (dBP). *Conclusions*. Our results demonstrate that a large number of T1D patients with hypertension do not receive appropriate treatment; few of the treated T1D patients achieved the target sBP and dBP values. Greater attention should be paid to blood pressure evaluation, hypertension diagnosis, and treatment of T1D patients in Brazil.

## 1. Introduction 

Type 1 diabetes mellitus (T1D) is a chronic disease that carries a great risk of morbidity and mortality, as a result of the microvascular and macrovascular complications that reduce an affected individual's quality of life and life expectancy [1]. Progress in diabetes management in recent decades has improved the survival rates among T1D patients, although life expectancy remains lower for these individuals compared to nondiabetic subjects of equal age [2].

Diabetes has emerged as a major health problem in societies in which noncommunicable diseases are the most common causes of disability and death [1, 2]. Furthermore, diabetes treatment has become a large financial burden because of the increased associated direct and indirect costs [3].

The occurrence of hypertension in T1D patients is directly correlated with the presence of microvascular complications, primarily nephropathy and retinopathy and the progression of these chronic complications [4, 5]. There is strong evidence, relating to the efficacy and cost effectiveness of treatment, to support blood pressure control in T1D and T2D patients, as well as the nondiabetic population, for reducing levels of morbidity and mortality [6–8]. Target blood pressure levels have been described in many guidelines, including the American Diabetes Association (ADA) [9], American Heart Association (AHA) [10], and Brazilian Diabetes Society (BDS) [11]. However, a large gap remains between the recommendations for blood pressure control and the values that have been described in most observational T1D [12–14] and T2D [15] studies.

Previous studies on the prevalence, awareness, treatment type, and control of hypertension have examined nondiabetic populations or T2D patients [16, 17] but rarely T1D patients. The Coronary Artery Calcification in type 1 Diabetes Study (CACT1) [12] demonstrated a higher rate of hypertension among T1D (43%) patients compared to nondiabetic subjects (15%) but observed a similar rate of hypertension awareness between T1D subjects (53%) and controls (45%). Furthermore, the EURODIAB study demonstrated a hypertension prevalence of 24% among T1D patients and less than half of these patients were aware of this condition. Only 42.2% of the T1D patients in this study received treatment, and only 26.7% of the treated T1D patients attained the established blood pressure targets [13]. 

The results of these studies emphasize the difficulties associated with the treatment of hypertensive T1D patients in routine clinical care and the need for improved treatment quality. 

The absence of national data on the prevalence, awareness, type of treatment, and control of hypertension in T1D patients led the Brazilian Type 1 Diabetes Study Group (BrazDiab1SG) to conduct this study, seeking to provide current and reliable data on the topic with regard to the ADA guidelines.

## 2. Research Design and Methods

This was a multicenter, cross-sectional, and observational study that was conducted between December 2008 and December 2010 in 28 public secondary (ambulatory outpatient clinics) and tertiary care level clinics (ambulatory outpatient clinics in university hospitals), located in 20 cities in four Brazilian geographic regions (north/northeast, midwest, southeast and south). The detailed data collection methods have been described previously [18]. Briefly, all patients received health care from the National Brazilian Health Care System (NBHCS). All eligible participating centers possessed a diabetes clinic with at least one endocrinologist. Each clinic provided data from a minimum of 50 consecutive outpatients with an initial diagnosis of T1D who regularly attended the clinic. The inclusion criteria consisted of a diagnosis of T1D by a physician that was based on the typical clinical presentation, including variable degrees of weight loss, polyuria, polydipsia, and polyphagia, and the need for continuous insulin use since T1D diagnosis. All patients were diagnosed between 1960 and 2010. 

The following variables were assessed in each interview during the clinical visit: current age, age at diagnosis, duration of diabetes (y), height (m), weight (kg), mean blood pressure (systolic and diastolic in mmHg from three consecutive measurements in one day using a standard clinical sphygmomanometer), modality of diabetic treatment, comorbidities, frequency of SBGM, and smoking status. The levels of glycated hemoglobin (HbA1c), fasting plasma glucose (FPG), total cholesterol, LDL cholesterol, HDL cholesterol, and triglycerides on the last clinical visit were obtained from medical records. The screenings for retinopathy, using fundoscopy; nephropathy, according to microalbuminuria; and foot examinations in patients with diabetes duration equal or greater than five years were noted when these procedures were performed within one year of the study assessment. 

Demographic, educational, and economic data were also obtained. Patients with diabetes for less than five years were not included in the analysis of diabetic chronic microvascular complications (*n* = 1, 160, 32.3%). Each local center's ethics committee approved the study (the appendix). The Brazilian Diabetes Society (BDS) monitored and reviewed all study-related documents and approved all amendments and publications. Each center's coordinator reviewed the chart form prior to final approval.

The following ADA goals for adequate metabolic and clinical control [11] were adopted by the BrazDiab1SG: HbA1c < 7.5% for T1D patients of 13 to 19 years of age, HbA1c < 8% for T1D patients of six to twelve years of age, HbA1c > 7.5% and < 8.5% for T1D for patients less than 6 years of age, and HbA1c < 7% for adult T1D patients; systolic blood pressure (sBP) < 130 mmHg; diastolic blood pressure (dBP) < 80 mmHg; body mass index (BMI) < 25 kg/m^2^; FPG < 130 mg/dL (7.2 mmol/L); total cholesterol < 200 mg/dL (5.2 mmol/L); HDL cholesterol > 40 mg/dL for men (1.1 mmol/L) and > 50 mg/dL (1.3 mmol/L) for women; LDL cholesterol < 100 mg/dL (2.6 mmol/L); non-HDL cholesterol < 130 mg/dL (3.30 mmol/L); and triglycerides < 150 mg/dL (1.7 mmol/L).

Hypertension in adults was defined as sBP ≥ 140 mmHg and/or dBP ≥ 90 mmHg, measured during the last clinical visit [8] or was self-reported, while hypertension in children and adolescents was defined as a sBP or dBP ≥ 95th percentile, according to the patient's age, sex,missimg and height [19] with the measurements taken during the clinical visit. Patient awareness of hypertension in adults was based on patient self-reporting of any prior hypertension diagnosis that was made by a health practitioner on at least two separate occasions. Patients who received angiotensin-converting enzyme (ACE) inhibitors or angiotensin receptor blockers (ARBs) for the treatment of micro- or macroalbuminuria and those who were not hypertensive were not included in the hypertensive group (*n* = 197, 7.5%). 

Microalbuminuria and clinical nephropathy were defined according to the ADA recommendations [9]. Overweight adults were defined as those with a BMI ≥ 25 kg/m^2^, and obesity was defined as a BMI ≥ 30 kg/m^2^ [9]. Overweight children and adolescents were defined as those with a BMI ≥ the 85th percentile, and obesity was defined as a BMI ≥ the 95th percentile, according to the patient's age and gender [20]. 

 HbA1c values obtained in the last clinical visit and the corresponding measurement methods were collected from the patients' medical charts. HbA1c measurements were obtained for 3,099 patients (86.2%), using methods that were certified by the National Glycohemoglobin Standardization Program (NGSP); of these, 1,766 patients (51.3%) were evaluated using high-performance liquid chromatography, whereas 1,601 patients (46.6%) were evaluated using turbidimetry. Measurements of HbA1c obtained using methods that were not certified by the NGSP, missing data, and HbA1c measurements obtained more than one year before the study assessment were excluded from the glycemic control analyses (*n* = 494, 13.8%). FPG, triglycerides, HDL, and total cholesterol were measured using enzymatic techniques. LDL levels were calculated using Friedewald's equation [21]. BMI (kg/m^2^) was determined by dividing an individual's weight (kg) by the square of his∖her height (m^2^). Current smoking was defined as smoking more than one cigarette per day at the time of the interview. Patients younger than 13 years of age were considered children (toddlers, preschoolers, or grade-schoolers), patients ≥ 13 years and ≤ 18 years were deemed adolescents, and patients > 18 years were considered adults [9]. 

### 2.1. Statistical Analysis

A detailed description of the study sample calculation has been given previously [18]. Briefly, the study sample represented the distribution of T1D cases across four geographic regions in Brazil. The proportion of cases from each region was estimated using the overall population distribution reported in the 2000 Brazilian Institute of Geography and Statistics Population Census (IBGE); 38.8%, 31.7%, 23.0%, and 6.6% of the population was distributed in the southeast, north/northeast, south, and midwest regions, respectively [22]. These data were combined with the national estimates of the prevalence of diabetes, which were derived from a 1988 survey, to determine the minimum number of patients to be studied in each region [23]. Recruitment in each region of the country enrolled > 95% of the estimated number of T1D patients for the region. Economic status was defined according to the Brazilian Economic Classification Criteria [24]. This classification also takes into account education level, which is categorized as illiterate/incomplete primary education, complete primary education/incomplete secondary education, complete secondary education/incomplete high school, complete high school/some college, or complete college education. The following classes of economic status were considered for this analysis: high, middle, low, and very low [24]. 

Data are presented as the means (± SD) for continuous variables and as counts (relative frequencies) for discrete variables. For analyzing blood pressure data, the mean from three consecutive measurements in a single day was used. Comparisons between numeric variables were performed using independent two-sided *t*-tests and two-sided *z*-tests for discrete variables with a normal approximation to the binomial distribution. An unadjusted Pearson's correlation coefficient was calculated when indicated. A multiple logistic regression (Forward-Wald) was performed with hypertension (yes/no) as the dependent variable. The following independent variables were included: race (Caucasian or non-Caucasian based on self-reporting), age, BMI, geographic region, gender, urine albumin excretion rate, and economic status. The Nagelkerke *R* square value was also calculated for this analysis. All of the analyses were performed using SPSS version 16.0 (Statistical Package of Social Sciences, Chicago, IL, USA). Odds ratios with 95% confidence intervals (CI) were performed when indicated. A two-sided *P* value less than 0.05 was considered significant.

## 3. Results

The clinical and demographic data for the study population are shown in Table 1. The majority of the patients evaluated were less than 30 years old (*n* = 1, 077, 30%). 

Due to missing data, in the total population of 3,591 patients, 268 (7.5%) could not be classified as either hypertensive or normotensive. Among the 3,323 (92.5%) T1D patients evaluated, a total of 689 (19.2%) of the studied patients were considered hypertensive, 236 (6.6%) were based on actual blood pressure measurements, and 453 (12.6%) were based on a history of or treatment for hypertension (self-reported). Hypertension was more frequent in adults than in children or adolescents (*n* = 562 (31.3%) versus *n* = 127 (8.3%), respectively, *P* < 0.001). Patients with hypertension were also older, exhibited longer duration of diabetes and had higher BMI, total cholesterol, triglycerides, LDL-C, and HDL-C values than patients without hypertension (*P* < 0.001 for all comparisons). These data are listed in Table 1. 

A greater number of children and adolescents had missing blood pressure data than did adults (258 (96.3%) versus 10 (3.7%), respectively, *P* < 0.001), and these data are indicated in Table 2.

The mean age at the time of hypertension diagnosis was 20 ± 10.3 years, and the self-reported duration of hypertension was 3 years (range < 1 to 44 years). Patients who were aware of their hypertension were older (*P* < 0.001) and exhibited higher sBP (*P* = 0.001) and fewer borderline sBP of 140 mmHg (*P* = 0.01) and borderline dBP of 90 mmHg (*P* = 0.02) compared to patients who were unaware. A total of 370 (53.7%) of the hypertensive patients received treatment. More patients aware of their hypertensive status received treatment than did patients who were unaware of their condition (*P* < 0.001). These data are presented in Table 3.

Higher SBP and dBP values were also observed in treated patients compared to untreated patients (sBP: 132.99 ± 19.4 versus 123.1 ± 21.2 mmHg, respectively, *P* < 0.001, and dBP: 83.00 ± 12.32 versus 79.12 ± 14.20 mmHg, respectively, *P* < 0.001). In total, 207 (55.9%) of the 370 treated patients were administered only one antihypertensive agent; of these, 161 (43.5%) patients used ACE inhibitors and 46 (12.4%) patients received monotherapy with calcium channel blockers (*n* = 5, 1.3%), beta blockers (*n* = 10, 2.7%), angiotensin receptor blockers (ARBs) (*n* = 15, 4.1%), or diuretics (*n* = 16, 4.3%). A total of 122 (33%) patients received two drugs in the following combinations: ACE inhibitors plus diuretics, ARBs, beta blockers or calcium channel blockers, and ARBs plus diuretics or calcium channel blockers. Forty-one (11.1%) patients received triple therapy with ACE inhibitors and diuretics plus ARBs, beta blockers or calcium channel blockers.

A total of 76 (22.9%) treated hypertensive patients achieved the targeted blood pressure range. The patients' sBP values correlated with age (*r* = 0.47, *P* = 0.001), diabetes duration (*r* = 0.41, *P* < 0.001), total insulin dose (*r* = −0.17, *P* < 0.001), AER (*r* = 0.16, *P* < 0.001), BMI (*r* = 0.44, *P* < 0.001), total cholesterol (*r* = 0.11, *P* = 0.001), triglycerides (*r* = 0.10, *P* = 0.001), HDL cholesterol (*r* = 0.05, *P* = 0.01) and LDL cholesterol (*r* = 0.07, *P* = 0.001). The dBP values correlated with age (*r* = 0.40, *P* = 0.001), diabetes duration (*r* = 0.32, *P* < 0.001), total insulin dose (*r* = −0.12, *P* < 0.001), AER (*r* = 0.16, *P* < 0.001), BMI (*r* = 0.38, *P* < 0.001), total cholesterol (*r* = 0.15, *P* = 0.001), triglycerides (*r* = 0.14, *P* < 0.001), HDL cholesterol (*r* = 0.05, *P* = 0.01), and LDL cholesterol (*r* = 0.07, *P* = 0.001).

Patients with proliferative retinopathy or nonproliferative retinopathy had higher sBP and dBP values than patients without retinopathy (sBP: 124.5 ± 20.6 versus 121.2 ± 19.2 versus 113.1 ± 15.6 mmHg, respectively, *P* < 0.001, and dBP: 78.5 ± 11.9 versus 76.9 ± 11.5 versus 72.5 ± 10.8 mmHg, respectively, *P* < 0.001). Additionally, patients with clinical nephropathy or microalbuminuria had higher sBP and dBP values than patients without nephropathy (sBP: 123.3 ± 21.1 versus 120.9 ± 17.9 versus 113.3 ± 15.8 mmHg, respectively, *P* < 0.001, and dBP: 78.7 ± 12.6 versus 76.6 ± 10.7 versus 72.5 ± 10.7 mmHg, respectively, *P* < 0.001).

 Multivariate logistic analysis revealed that hypertension was directly associated with age (OR = 1.06; 95% CI (1.05–1.076; *P* < 0.001)), BMI (OR = 1.13; 95% CI (OR = 1.09–1.17; *P* < 0.001)), AER level [OR = 1.02; 95% CI (1.01–1.03; *P* < 0.001)] and male gender [OR = 1.35; 95% CI (1.02–1.80; *P* < 0.001)]. Caucasian race was also associated with a lower odds ratio of hypertension (OR = 0.68; 95% CI (0.51–0.91; *P* = 0.01)). This model described 25.3% of the probability of hypertension for a given patient.

## 4. Discussion

This study demonstrated that, while nearly 20% of the patients examined exhibited hypertension, only 53.7% of these patients received treatment. Moreover, only 22.3% of the treated hypertensive patients achieved the targeted sBP and dBP values. Hypertension was more common in non-Caucasian adults and was associated with microvascular complications and other cardiovascular risk factors, such as being overweight or obese and exhibiting dyslipidemia. 

The ADA provides recommended blood pressure levels for all diabetic patients, but approximately 7.5% of the patients participating in the current study received no such evaluation in the year prior to the study. This was commonly observed primarily in children and adolescents, as well as individuals from the north/northeast and midwest regions of Brazil. Some diabetes clinical care centers in Brazil may not include blood pressure evaluations in their routine care of children and adolescents. Although hypertension was more frequent among adults (31.3%), in our diabetic study population, 8.3% of diabetic children and adolescents also were hypertensive. Few studies of hypertension in T1D patients have been conducted; the majority of these studies analyzed hypertension in adult diabetic patients and reported a prevalence of 24 to 43% [12–14, 25–29], which is similar to those observed in the current study. In addition, an observational study in Rio de Janeiro, Brazil, identified a hypertension prevalence of 6.8% in nondiabetic children and adolescents [30]. Our data on children and adolescents are similar to those published by the Search Study (5.9%) [26], although our prevalence figures were higher than those published (4%) in a recent Norwegian study [29]. Studies of elevated sBP or dBP in children and adolescents (greater than the 90th percentile for age, gender, and height) have reported a prevalence of hypertension between 6% and 23%, depending on the presence of other cardiovascular risk factors [26–29]. Additionally, the prevalence of hypertension has been shown to increase fourfold in overweight or obese children and adolescents [29]. Age, diabetes duration, the presence of chronic complications, race, and the number of medical visits with available blood pressure evaluations may account for the differences between our study and those conducted previously.

More than one-third of our patients who were unaware of their hypertensive condition were children and adolescents. Importantly, all of these patients were treated by an endocrinologist in secondary and tertiary care settings.

Diabetes treatment in public clinics is financed by the NBHCS, and our data reveal that factors other than medical recommendations might likely interfere with diabetes care in Brazil [18, 31].

The guidelines recommend aggressive hypertension treatment in T1D patients, but only 53.7% of our patients received such treatment; similar results were described in the EURODIAB study [13]. In the current study, the majority of treated patients (55.9%) received only one antihypertensive drug, whereas 44.1% received two or more drugs. These results are in contrast with the results of previous studies reporting that up to 19% of T1D patients received two antihypertensive agents [12, 13]. In addition, only 11.1% of the patients in the current study received triple therapy, which is higher than the percentage described in the CACT1 (7%) and EURODIAB (1.9%) studies. The abovementioned studies were conducted 5 to 10 years before our study, suggesting that an increase in the intensity of hypertension treatment has occurred in recent years, as previously observed in a temporal analysis of EURODIAB [32]. However, less than one-third of our patients and T1D patients in the EURODIAB study [13] exhibited controlled sBP and dBP levels, which suggests that factors beyond pharmacological treatment might influence blood pressure control. Additionally, compared to our study, a larger percentage of the CACT1 patients (up to 64%) exhibited controlled blood pressure levels [12]. This difference may be attributed to study design, as the patients in the EURODIAB and our corresponding studies were not volunteers.

The Pittsburgh Epidemiology of Diabetes Complications Study utilized different targets for blood pressure and demonstrated small improvements in hypertension control, primarily in younger-aged groups of T1D patients, over a 10-year follow-up period [33]. One study that was performed at academic medical centers observed a low rate of medication management when T1D patients remained above their blood pressure goal [34]. As factors such as hypertension, obesity, and being overweight are indicators of CVD risk, we concluded that the young patients that were evaluated represent a high-risk group for the development of microvascular and macrovascular complications associated with diabetes, as described previously [4, 5, 26–28]. Furthermore, our study demonstrated a clear association between the different stages of retinopathy and nephropathy and increasing levels of blood pressure. 

The BrazDiab1SG is the only national registry on the prevalence, awareness, and treatment of hypertension in T1D; the principal strength of our study is our large sample size, which included a representative sample of T1D distribution in the young Brazilian population. Importantly, our study included patients from a wide range of racial backgrounds from all geographic regions of the country, and it maintained a uniform, standard recruitment protocol at all of the participating centers. 

However, several limitations of the current study must be addressed. We used a clinical definition of T1D that was assigned by physicians and was applicable to all patients, which is similar to previous studies [15, 16]. However, autoantibody and C-peptide levels were not measured. Therefore, some patients with other types of diabetes may have been included. Nevertheless, it is important to emphasize that 93.1% of our patients were diagnosed before the age of 30, which supports the high probability that these patients had T1D. Also, as all of the patients in this study lived in large cities and were seen in a public center by a specialist, patients who relied on primary care facilities and lived in rural areas may have been overlooked. Although 14% of the Brazilian population lives in rural areas, the prevalence of T1D in this group is very low [34], and consequently rural T1D patients represent the minority of patients who receive treatment in Brazil. Additionally, patients recruitment within each center may have produced a selection bias for age because the majority of our patients were younger than 30 years of age. Moreover, there were missing data for blood pressure measurements, which were primarily observed in the youngest patients. Additionally, the prevalence of hypertension may have been overestimated because diagnosis was based on the measurement of a blood pressure in one day rather than two separate measurements on two separate days. Although we used a standard clinical sphygmomanometer, the possibility for misclassification remains, especially, at borderline diagnosis levels for sBP and dBP. Misclassification was noted in our sample in the analysis of the lack of hypertension awareness, which was more frequent in the borderline group of patients. The use of self-reported hypertension as a criterion for awareness may have also produced a bias in the diagnosis of this condition. 

Therefore, to our knowledge, this research constitutes the first national report on the prevalence of hypertension in T1D in Brazil, a disease with increasing incidence in our country [35]. Our results demonstrate that many T1D patients with hypertension do not receive antihypertensive treatment; moreover, few treated T1D patients receive combined therapy, and few of these patients achieve their targeted sBP and dBP values. The evaluation of blood pressure in children and adolescents is likely not included in all routine diabetic clinical care centers. Thus, greater attention should be paid to blood pressure evaluation and hypertension diagnosis and treatment for T1D patients in Brazil. 

## Figures and Tables

**Table 1:** Demographic data of the studied population.

Variable	
Age, years	21.2 ± 11.7
Gender, female (%)	2,010 (56.0)
Age at diagnosis, years	10.0 (<1 to 44)
Age at diagnosis, years (%)	
0–4.9	667 (18.5)
5–9.9	961 (26.8)
10–14.9	941 (26.2)
≥15	1,022 (28.5)
Diabetes duration, years	9.6 ± 8.1
Diabetes duration, years (%)	
0–4.9	672 (18.7)
5–9.9	961 (26.8)
10–14.9	941 (26.2)
≥15	1,017 (28.3)
Level of care, *n* (%)	
Secondary	995 (27.7)
Tertiary	2,596 (72.3)
Geographic region (%)	
Southeast	1,424 (39.7)
North/northeast	1,113 (31)
South	820 (22.8)
Mid-west	234 (6.5)

The data are presented as counts (percentage), means ± SD, or medians (minimum/maximum). *African-Brazilians, Mulattos, Asians, and Native Indians; **Data were available for 3,434 patients.

**Table 2:** Demographic, clinical, and laboratory data for the presence of hypertension in the studied population.

Variables	Hypertension*		
	Yes (%)	No (%)	*P* value
*n* (%)	689 (19.2)	2,634 (73.4)	—
Age, years	30.5 ± 12.8	19.7 ± 10.4	<0.001
Age at diagnosis of diabetes, years	14.8 ± 9.1	11.2 ± 7.6	0.19
Gender, female *n* (%)	318 (21.9)	1,136 (78.1)	0.19
Duration of diabetes, years	15.7 ± 9.6	8.5 ± 7.0	<0.001
Race, *n* (%)			<0.001
Caucasian	353 (18.2)	1,585 (81.8)	
Non-Caucasian	336 (24.3)	1,049 (75.7)	
Economic status, *n* (%)**			0.02
High	62 (26.6)	171 (73.4)	
Medium	174 (23.2)	575 (76.8)	
Low	208 (18.9)	890 (81.1)	
Very low	242 (21.3)	896 (78.6)	
BMI (Kg/m^2^)	24.0 ± 4.7	21.4 ± 3.9	<0.001
Overweight/obesity, *n* (%)	276 (40.5)	767 (29.5)	<0.001
Fasting glycemia (mg/dL)	179.7 ± 107.1	182.5 ± 105.7	0.5
HbA1c (%)	9.3 ± 2.4	9.3 ± 2.3	0.8
HbA1c < 7%, *n* (%)	77 (12.8)	294 (13.0)	0.7
sBP (mmHg)	128.4 ± 20.7	107.9 ± 12.7	<0.001
dBP (mmHg)	81.31 ± 3.3	69.5 ± 9.4	<0.001
Total cholesterol (mg/dL)	178.5 ± 48.0	168.7 ± 39.6	<0.001
Triglycerides (mg/dL)	110.5 ± 85.4	89.0 ± 63.6	<0.001
HDL cholesterol (mg/dL)	53.4 ± 16.9	52.5 ± 14.0	<0.001
LDL cholesterol (mg/dL)	104.3 ± 39.7	99.0 ± 31.5	0.001
Current smoker, y (%)	31 (4.5)	110 (4.2)	0.9
Insulin dose (U/Kg/day)	0.83 ± 0.36	0.93 ± 0.39	<0.001
Number of clinical visits (previous year)	4.07 ± 1.7	4.10 ± 1.6	0.6

*Missing cases 268 (7.5%).

**Missing cases 130 (3.6%).

BMI: body mass index; sBP: systolic blood pressure; dBP: diastolic blood pressure; HDL: high-density lipoprotein; LDL: low-density lipoprotein.

Overweight/obesity were considered together.

The data are presented as counts (percentage) or means ± SD; *African-Brazilians, Mulattos, Asians, and Native Indians.

**Table 3:** Hypertension awareness.

Variables	Hypertension awareness		
	Yes (%)	No (%)	*P* value
*n* (%)	453 (65.7)	236 (34.3)	—
Children and adolescents, *n* (%)	28 (22)	99 (78)	<0.001*
Adults, *n* (%)	425 (75.6)	137 (24.4)	
sBP (mmHg)	129.8 ± 20.1	124.1 ± 21.9	0.001
dBP (mmHg)	81.5 ± 12.4	80.1 ± 15.2	0.1
Borderline sBP (140 mmHg) (%)	11	17.4	0.01
Borderline dBP (90 mmHg) (%)	17	28.4	0.01
Antihypertensive treatment (%)	67.3	11.8	<0.001

*P < 0.001 (children and adolescents versus adults).

sBP: systolic blood pressure; dBP: diastolic blood pressure.

The data are presented as counts (percentage) or means ± SD.

## Appendix

### *Brazilian Type 1 Diabetes Study Group (BrazDiab1SG)

*Executive Steering Committee: Marilia Brito Gomes (Chair), Roberta Cobas, Sergio Atala Dib and Carlos Negrato*. Universidade Estado Rio de Janeiro: Roberta Cobas*, Alessandra Matheus, Lucianne Tannus; Universidade Federal Rio de Janeiro: Lenita Zajdenverg*, Melanie Rodacki; Hospital Geral de Bonsucesso: Neuza Braga Campos de Araújo*, Marilena de Menezes Cordeiro; Hospital Universitário Clementino Fraga Filho—IPPMG: Dr. Jorge Luiz Luescher*; Renata Szundy Berardo; Serviço de Diabetes da Disciplina de Endocrinologia e Metabologia do Hospital das Clínicas da Universidade de São Paulo: Marcia Nery*; Catarina Cani; Maria do Carmo Arruda Marques; Unidade de Endocrinologia Pediátrica da Santa Casa de Misericórdia de São Paulo: Luiz Eduardo Calliari*, Renata Maria de Noronha; Instituto da Criança do Hospital das Clínicas da Universidade de São Paulo: Thais Della Manna*, Roberta Salvodelli, Fernanda Garcia Penha; Hospital das Clínicas da Faculdade de Medicina de Ribeirão Preto—USP: Milton Cesar Foss*, Maria Cristina Foss-Freitas; Ambulatório da Faculdade Estadual de Medicina de São José do Rio Preto: Antonio Carlos Pires*, Fernando Cesar Robles; Associação de Diabéticos de Bauru: Carlos Antonio Negrato*, Maria de Fatima Guedes; Centro de Diabetes da Escola Paulista de Medicina: Sergio Atala Dib*, Patricia Dualib; Clínica de Endocrinologia da Santa Casa de Belo Horizonte Setor Diabetes Tipo 1: Saulo Cavalcanti da Silva*, Janice Sepulveda; Ambulatório Multiprofissional de Atendimento à Diabetes do Hospital de Clínicas da Universidade Estadual de Londrina: Henriqueta Guidio de Almeida*, Emerson Sampaio; Hospital de Clínicas da Universidade Federal do Paraná: Rosangela Roginski Rea*, Ana Cristina Ravazzani de Almeida Faria; Instituto da Criança com Diabete Rio Grande Sul: Balduino Tschiedel*, Suzana Lavigne, Gustavo Adolfo Cardozo; Hospital de Clínicas de Porto Alegre: Mirela Azevedo*, Luis Henrique Canani, Alessandra Teixeira Zucatti; Hospital Universitário de Santa Catarina: Marisa Helena Cesar Coral*, Daniela Aline Pereira; Instituto de Diabetes-Endocrinologia de Joinville: Luiz Antonio de Araujo*; Hospital Regional de Taguatinga, Brasília: Hermelinda Cordeiro Pedrosa*, Monica Tolentino; Flaviene Alves Prado; Hospital Geral de Goiânia: Dr Alberto Rassi: Nelson Rassi*, Leticia Bretones de Araujo; Centro de Diabetes e Endocrinologia do Estado da Bahia: Reine Marie Chaves Fonseca*; Alexis Dourado Guedes, Odelisa Silva de Mattos; Universidade Federal do Maranhão: Manuel Faria*, Rossana Azulay; Centro Integrado de Diabetes e Hipertensão do Ceará: Adriana Costa e Forti∗, Maria Cristina Façanha; Universidade Federal do Ceará: Renan Montenegro Junior*, Ana Paula Montenegro; Universidade Federal de Sergipe: Naira Horta Melo*, Karla Freire Rezende; Hospital Universitário Alcides Carneiro: Alberto Ramos*; Hospital Universitário João de Barros Barreto, Pará: João Soares Felicio*, Flavia Marques Santos; Hospital Universitário Getúlio Vargas, Hospital Adriano Jorge: Deborah Laredo Jezini*.

## Abbreviations

ADA: American Diabetes Association
CV: Cardiovascular
T1D: Type 1 diabetes
BDS: Brazilian Diabetes Society
sBP: Systolic blood pressure
dBP: Diastolic blood pressure
BMI: Body mass index
HbA1C: Glycated hemoglobin
T2D: Type 2 diabetes
FBG: Fasting blood glucose
NBHCS: National Brazilian Health Care System
BrazDiab1SG: Brazilian Type 1 Diabetes Study Group,
SBGM: Self-blood glucose monitoring
HDL: High-density lipoprotein
LDL: Low-density lipoprotein
NGSP: National Glycohemoglobin Standardization Program
ACE: Angiotensin-converting enzyme
ARBs: Angiotensin receptor blockers
AER: Albumin excretion rate.
